# Phase II study of single agent capecitabine in the treatment of metastatic non-pancreatic neuroendocrine tumours

**DOI:** 10.1038/bjc.2011.76

**Published:** 2011-03-08

**Authors:** L Medley, A N Morel, D Farrugia, N Reed, N Hayward, J M Davies, O Kirichek, R V Thakker, D C Talbot

**Affiliations:** 1 University Department of Medical Oncology, Cancer and Haematology Centre, Churchill Hospital, Headington OX3 7LJ, UK; 2 Gloucestershire Oncology Centre, Cheltenham General Hospital, Cheltenham GLOS GL53 7AL, UK; 3 Beatson Oncology Centre, Gartnavel General Hospital, Glasgow G12 OYN, UK; 4 Academic Endocrine Unit, Nuffield Department of Medicine, Oxford Centre for Diabetes, Endocrinology and Metabolism, University of Oxford, Churchill Hospital, Headington OX3 7LJ, UK

**Keywords:** neuroendocrine tumour, capecitabine, 5-fluorouracil

## Abstract

**Background:**

This study sought to determine the safety of single agent capecitabine, a pro-drug of 5FU, in patients with metastatic non-pancreatic neuroendocrine tumours (NETs).

**Methods:**

Multicentre phase II, first-line study design. Oral capecitabine was administered on days 1–14 of 3-week cycles.

**Results:**

Treatment was safe and well tolerated. Common toxicities were diarrhoea and fatigue.

**Conclusion:**

The study provides evidence to support the use of capecitabine as a substitute for infusional 5FU in the management of NETs.

Neuroendocrine tumours (NETs) arise from secretory cells in the nervous and endocrine systems that often present with symptoms related to the excess production of hormones, neuropeptides and neurotransmitters (Oberg and Jelic, 2009). The incidence increased from 1.1 to 5.2 cases per 100 000 between 1973 and 2004, in part reflecting better detection and diagnosis (Yao *et al*, 2008).

For unresectable tumours, the choice of treatment is determined by the anatomical origin, degree of differentiation and endocrine function of the NET (Plockinger, 2007; Modlin *et al*, 2008; Clark *et al*, 2009). Therapeutic options include somatostatin analogues, targeted radiotherapy, immunotherapy (interferon-*α* (IFN-*α*)), hepatic artery embolisation, radiofrequency ablation, cytotoxic chemotherapy and agents that target mTOR or angiogenesis (Modlin *et al*, 2008; Basu *et al*, 2010). For patients with advanced pancreatic NETs, streptozotocin (STZ)-based chemotherapy regimens are the standard of care (Moertel *et al*, 1992). Streptozotocin-based therapy also has a role in the treatment of non-pancreatic NETs, although these tumours tend to be less sensitive. The pyrimidine analogue 5-Fluorouracil (5FU) is often used in combination with STZ (Basu *et al*, 2010), but toxicity remains an issue, highlighting the need to improve tolerability without affecting efficacy.

Treatment of colorectal cancer (CRC) with capecitabine, the orally administered pro-drug of 5FU, results in better clinical benefit compared with bolus 5FU (Hoff *et al*, 2001; Cassidy *et al*, 2002). Meta-analysis has demonstrated increased response rates following prolonged administration of 5FU compared with bolus 5FU (Meta-analysis Group in Cancer, 1998). In NETs, infusional 5FU combined with IFN*α* resulted in clinical activity and good tolerability (Andreyev *et al*, 1995). We report here the results of a study of capecitabine monotherapy in metastatic non-pancreatic NETs. Our aims were to evaluate the tolerability, clinical outcome and to establish the rationale for using capecitabine in combination therapy as an alternative to infusional 5FU in NETs.

## Patients and methods

This was an open-label, multi-centre, single arm, phase II study. Patients received oral capecitabine 2000 mg m^−2^ per day administered in two divided doses for 14 consecutive days every 3 weeks. Patients with ⩾grade II NCI-CTC toxicity had treatment withheld until toxicity returned to ⩽grade I. For second and third occurrences of grade II, and first or second episodes of grade III, toxicity doses were reduced by 25 and 50%, respectively. Treatment was discontinued in cases of subsequent toxicity or disease progression. Baseline evaluations included: complete medical history, physical and radiological examinations, full blood count, biochemistry, Chromogranin A and urinary 5-HIAA. Toxicity was evaluated every 3 weeks. Response was evaluated every 9 weeks according to RECIST criteria. Biochemical responses were defined as: complete response (CR), return of both markers to normal; partial response (PR), >50% decrease from baseline; stable disease (SD), <50% decrease and <25% increase from the baseline; progressive disease (PD), >25% increase from the baseline. Overall response was defined as either the best radiological or biochemical response obtained.

Adult patients with a histological diagnosis of inoperable, locally advanced or metastatic NET were eligible for inclusion if they had: biochemically or radiologically measurable disease, an ECOG performance status of 0–2, adequate organ function (white blood cells ⩾3 × 10^9^ l^−1^, neutrophils ⩾1.5 × 10^9^ l^−1^, platelets ⩾100 × 10^9^ l^−1^, bilirubin ⩽30 *μ*mol l^−1^, creatinine ⩽150 *μ*mol l^−1^) and life expectancy of at least 3 months. Patients with stable disease on octreotide therapy before study entry were allowed to continue octreotide. Patients were excluded if they had a diagnosis of pancreatic NET, Ki67>2%, had received previous chemotherapy, previous malignancy or uncontrolled systemic disease. Local Research Ethics Committees approved the study and written informed consent was obtained from all patients. The study was carried out in accordance with Good Clinical Practice.

### Statistical considerations

Gehan's two-stage design was adopted in this study to allow for the early rejection of an ineffective drug. The target patient accrual for the first stage was 19 patients with additional patients recruited depending on response rate such that if capecitabine were active in more than 15% of patients then the probability of it being rejected erroneously would have been 0.046 (Gehan, 1961). Changes in the severity of diarrhoea were analysed by comparing baseline levels with those of each subsequent cycle using the Stuart–Maxwell test. Overall survival was estimated using the Kaplan–Meier method.

## Results

A total of 20 patients were recruited between three sites in the UK (Oxford, Glasgow, Cheltenham) and followed up for a median of 27 months. One patient was replaced because of non-compliance with the study protocol. In total, 19 patients (demographics shown in Table 1) completed at least one cycle of treatment and were assessable for response and safety evaluation. Two patients remain on study, 4 years after enrolment.

Capecitabine was generally well tolerated (Table 2). Single episodes of grade IV toxicity were recorded for infection, raised liver function tests (LFT's), thrombocytopenia, leucopenia and neutropenia. The most common grade III toxicities were diarrhoea (26% patients), fatigue (21% patients) and raised LFTs (21% patients). Most patients, as expected, experienced a rash and/or hand-foot syndrome (HFS), but all were of grade I-II toxicity. The Stuart–Maxwell test showed no statistically significant increase in the grade of diarrhoea between baseline and subsequent cycles of capecitabine therapy.

A total of 13 (68%) patients achieved a radiological SD of whom two (11%) also had biochemical PR by Chromogranin A. Three (16%) patients had radiological PD and three (16%) were not evaluable radiologically. There were no radiological PR or CR, thus the overall disease control rate was 68%. Four (21%) patients maintained SD for >12 months. Of these, two remain well in the follow-up phase of study and two discontinued treatment because of toxicity. Overall seven patients came off study due to PD (37%), five due to toxicity (26%), three by patient choice and two by investigator choice. The investigators’ reason in both cases was to defer treatment until symptomatic disease progression.

The median PFS was 9.9 months (lower quartile: 4.4, upper quartile: 36.7) and median OS was 36.5 months (lower quartile: 19.9, upper quartile not yet reached).

## Discussion

We report that capecitabine monotherapy in metastatic non-pancreatic NETs is safe and well tolerated. The toxicity experienced was similar to that reported in CRC (Hoff *et al*, 2001; Cassidy *et al*, 2002; Saif *et al*, 2008). We observed lower grades of HFS in patients with NETs compared with CRC and diarrhoea, both a feature of carcinoid syndrome and capecitabine toxicity, was not significantly exacerbated compared with baseline. Higher rates of grade III–IV diarrhoea were observed in this study relative to studies of capecitabine in CRC (Cassidy *et al*, 2002; Saif *et al*, 2008), which may be disease related. In CRC, there are clinically significant differences in the tolerability of capecitabine over bolus 5FU (Meta-analysis Group in Cancer, 1998; Hoff *et al*, 2001). Although uncomplicated hyperbilirubinemia and HFS occur more frequently with capecitabine, patients benefit from decreased rates of stomatitis, alopecia, neutropenia and neutropenic fever and sepsis (Van Cutsem *et al*, 2001; Cassidy *et al*, 2002). Moreover, in rectal cancer, capecitabine had superior or equivalent efficacy to metronomic 5FU infusion (Das *et al*, 2005; Saif *et al*, 2008). We show that capecitabine has an acceptable safety profile in non-pancreatic NETs justifying its inclusion in current and novel combination regimens as a substitute for conventional infusional 5FU.

Our study excluded patients for whom combination chemotherapy was indicated thus high-response rates were not anticipated for this group of patients. Of three patients who had received octreotide for over 12 months before entry to the study two had stable disease following capecitabine that may have been the effect of ongoing octreotide therapy (Brizzi *et al*, 2009; Rinke *et al*, 2009). Although the study was designed to detect inactivity, it was not powered sufficiently to confirm efficacy. Thus, we can conclude that the disease control rate of 68%, two biochemical PRs and median OS of 36.5 months in this study only suggest that capecitabine may have antitumour activity in non-pancreatic NETs. These findings should be considered in the light of the PROMID Study (Rinke *et al*, 2009) that reported a 6 months (95% CI, 3.7–9.4 months) median time to tumour progression in the placebo-treated group. A randomised placebo-controlled trial including an octreotide treatment arm would be required to test this hypothesis.

Neuroendocrine tumours do not overexpress thymidine phosphorylase (TP), a key enzyme in activation of capecitabine to 5FU (Ishikawa *et al*, 1998). However, expression of thymidylate synthase (TS), an enzyme targeted by 5FU, is increased in poorly differentiated NETs (Ceppi *et al*, 2008). Infusional 5FU and oral agents, which give similar continuous exposure are thought to exert antitumour effect through TS inhibition, as opposed to short exposure to bolus 5FU, which targets RNA synthesis (Aschele *et al*, 1992). This strengthens the rationale for the use of infusional or continuous oral treatment schedules in NET therapy.

We have demonstrated the safety and tolerability of capecitabine in the setting of advanced non-pancreatic NETs. This study provides the rationale for inclusion of capecitabine in the UK NET*work* randomised phase II trial comparing capecitabine+STZ with capecitabine+STZ+cisplatin in patients with advanced NETs.

## Figures and Tables

**Table 1:** Patient characteristics

**Characteristic**	**No. of patients (%)**
Total evaluable	19
Median age at diagnosis (range)	63 (46–79)
	
*Gender*	
Male	10 (53)
Female	9 (47)
	
*ECOG performance status*	
0–1	16 (84)
2–3	1 (5)
Not recorded/available	2 (11)
	
*Site of primary NET*	
Gut	12 (63)
Ovary	1 (5)
Unknown	6 (32)
	
*Octreotide treatment*	
Not started	11 (58)
Started before study entry	8 (42)
	
*Site of metastasis at diagnosis (six patients had liver only metastasis, 11 multiple metastatic sites)*	
Liver	14
Mesentery, mesenteric nodes	5
Retroperitoneum	4
Peritoneum	2
Neck nodes	2
Iliac nodes	2
Pleura, lung	2
Other (breast, adrenal, pancreas, bone, ovary and coeliac nodes)	6

Abbreviation: NET=neuroendocrine tumour.

**Table 2:** Toxicity profile

**Side effects**	**Grade III toxicity number of Pts. (%)**
Diarrhoea	5 (26)
Fatigue	4 (21)
Infection	2 (11)^a^
Nausea	2 (11)
Vomiting	1 (5)
Pain	2 (11)
Hand-foot syndrome	0 (0)
Cough	0 (0)
Rash	0 (0)
Loss of appetite	1 (5)
Stomatitis	0 (0)
Flushes	0 (0)
Headache	0 (0)
Bleeding	0 (0)
Neuropathy	0 (0)
	
*Laboratory*	
Abnormal LFT	4 (21)^a^
Hyponatraemia	0 (0)
Hypokalaemia	2 (11)
Urea	1 (5)
Haemoglobin	1 (5)
Platelets	1 (5)^a^
White cell count	1 (5)^a^
Neutropenia	1 (5)^a^

Abbreviation: LFT=liver function test.

a One of these was grade IV.
